# Gadofosveset-Trinatrium-Enhanced MR Angiography and MR Venography in the Diagnosis of Venous Thromboembolic Disease: A Single-Center Cohort Study

**DOI:** 10.3390/diseases10040122

**Published:** 2022-12-05

**Authors:** Manuela A. Aschauer, Ingeborg M. Keeling, Carmen V. Salvan-Schaschl, Igor Knez, Barbara Binder, Reinhard B. Raggam, Ameli E. Trantina-Yates

**Affiliations:** 1 Department of Radiology, Medical University of Graz, 8036 Graz, Austria; 2 Department of Cardiac Surgery, Medical University of Graz, 8036 Graz, Austria; 3 Department of Dermatology, Medical University of Graz, 8036 Graz, Austria; 4 Department of Angiology, Medical University of Graz, 8036 Graz, Austria

**Keywords:** magnetic resonance imaging, magnetic resonance angiography, magnetic resonance venography, gadolinium-containing contrast agent, pulmonary artery embolism, deep vein thrombosis

## Abstract

Background: The aim of this single-center combined prospective/retrospective cohort study was to analyze Gadolinium (Gd)-enhanced MRA (magnetic resonance angiography) and MRV (MR venography) for the diagnosis of pulmonary artery embolism and deep venous thrombosis. The gold standard methods result in major exposure to radiation and a high amount of nephrotoxic iodinated contrast media. This is the first larger contrast-enhanced MR imaging study of acute and chronic venous thromboembolic disease of various stages. Methods: We prospectively examined 88 patients presenting clinical signs of deep vein thrombosis and/or pulmonary artery embolism. A single-session, one-stop shop Gd-enhanced MRA/MRV at 1.5 Tesla, using gradient echo sequences with very short repetition and echo times as well as low flip angles with subtraction and three-dimensional reconstruction, was performed. A diagnosis was made with the consensus of two experienced radiologists. Results: We observed excellent MRA image quality in 87% and even higher diagnostic image quality of MRV in 90% of our examinations. Pulmonary artery embolism occurred with deep vein thrombosis in 22%. Conclusions: Gd-enhanced MRA/MRV provided excellent image quality for the diagnosis of venous thromboembolic disease in the majority of cases. It may be particularly useful to plan and follow-up filter implantation and retrieval in the inferior caval vein.

## 1. Introduction

Deep vein thrombosis, the major cause of venous thromboembolic disease (VTE), occurs with an incidence of roughly 1.5% per 1000 persons per year [1]. VTE is a serious, or even fatal, medical condition both in its acute and chronic form. It exhibits high morbidity and mortality depending on the stage of the disease, and it is responsible for a high number of annual hospital admissions [2,3,4,5]. Both pulmonary embolism (PE) and deep venous thrombosis (DVT) are manifestations of VTE and require immediate accurate diagnostic and therapeutic management [6,7]. A variety of imaging modalities are applied for the diagnosis of these diseases. The aim of our study was to analyze the performance of contrast-enhanced magnetic resonance angiography (MRA) and magnetic resonance venography (MRV) using a Gadolinium (Gd)-containing contrast agent and to evaluate its possible advantages in diagnosis including the location, morphology, extent and degree of VTE. The hypothesis of this study was that contrast-enhanced MRA/MRV (CE-MRA/MRV) uses a lower amount of contrast agent and less investigative resources for a complete work-up of possible PAE and TVT in a patient with suspected VTE. Furthermore, the therapeutic option of stent implantation can be evaluated in the same procedure of CE-MRA/MRV. It might be advantageous to give a contrast agent once for MRI and perform MRA for possible PAE and MRV for possible TVT, thus using a single dose of contrast agent and a single‚ ‘all-in-one‘ examination resource. Instead, two examinations, specifically duplex sonography and CTA, would be necessary. CTA needs a high amount of jodine-containing contrast agent and also a high amount of irradiation and time. Furthermore, duplex of the pelvis and the VCI are often limited due to inconclusiveness, especially in obese patients. In addition, duplex examinations are very investigator-dependent. A further limitation of CTA/CTV is that they suffer from incorrect timing and the rapid wash-out of the contrast agent from the arterial and venous system into the interstitial space. Conversely, Gadofosveset and other gadolinium-containing agents differ in retention. Gadofosveset has a longer half-life time in blood (blood pool agent).

## 2. Materials and Methods

We analyzed 92 combined MRA/MRV examinations of 50 female and 38 male patients (median age 55 years, range 18–91 years) performed over a period of 3 years. Four patients were analyzed twice for treatment control and/or the pre-evaluation of possible filter retrieval. All patients presented clinical signs of either PE and DVT, or suspected PE with known DVT, or suspected DVT with known PE. Informed consent was obtained from all patients. Ethical board approval was granted.

The recommended dose of the Gd-containing contrast medium Gadofosveset (Gdfos, Vasovist^®^, Bayer Pharma AG, Berlin, Germany, 0.03 mmol/kg body weight) was administered at a flow rate of 1 mL/sec and with a 30 mL saline flush for “one-stop shop” enhanced MRA of the pulmonary arteries and MRV for the peripheral veins in one session with 1.5 T Siemens Magnetom Symphony Maestro Class^®^ (4VA15A quantum gradients) (Siemens AG, Erlangen, Germany) [8]. A body-array flex coil was used for MRA of the pulmonary arteries. MRV was performed with a dedicated peripheral angiographic coil using a stepping-table technique as in peripheral arterial occlusive disease [8,9]. The patients were asked to hold their breath during a specific time of examination of the pulmonary arteries and the abdomen. The protocol details are shown in Table 1. Specifically, the table time was below 20 min in all patients. In this study, we used a gadolinium-based chelate and blood pool contrast agent, which resulted in an excellent resolution in the steady state phase and allowed for precise imaging in a single-dose protocol. Compared to conventional MRI agents, this contrast medium has the advantage of preferred serum albumin binding and a subsequently prolonged intravascular half-life, which enables vessel structure analysis and whole-body 3D-imaging in a single examination [8,9].

Image quality was evaluated independently and with a final consensus from two experienced radiologists. Subtraction, 3D maximum intensity projection (MIP) reconstruction and spin-reconstruction (180°) were used for the post-processing of our data on pulmonary arteries. Subtraction and 3D MIP reconstruction were conducted automatically; only spin-reconstruction (180°) was carried out by the technician.

The pulmonary trunk, the main right and left pulmonary artery as well as all lobular and segmental branches were analyzed. As agreed between two senior radiologists, image quality was considered excellent when the main artery, lobar and segmental bilateral arteries were present for assessment. The quality of the images was considered good if most segmental arteries were visible, moderate if the pulmonary trunk and the lobar arteries could be seen and poor if only the pulmonary trunk and the left and right main pulmonary arteries were present for interpretation. For peripheral veins, the examination included the inferior caval vein, the right and left renal veins, the bilateral common, external and internal iliac veins, the common femoral veins, the superficial and profound femoral veins, the popliteal veins, the anterior, posterior tibial veins and the peroneal veins. Furthermore, this study aimed at investigating the possible advantage of MRA/MRV to analyze vessels of the lower extremities, since the complete visualization of these vessels may be difficult to achieve using other methods. Image quality was considered excellent when all of the veins named above were present for interpretation, good when the veins including and proximal to the popliteal veins were visible and poor when only the inferior vena cava and the iliac veins could be interpreted while the femoral vein could be seen only insufficiently. A diagnosis was based upon the morphology (vessel lumen, wall and valves) of the vessels of all sizes, anatomical variants of vessels and presence, the location and extent of the thrombus as well as the acute or chronic aspect of the thrombosis. Specifically, a fresh thrombus was characterized by the absence of intraluminal contrast. A chronic thrombus presented partial recanalization, irregular filling defects, contour irregularity of the vessel wall, small thrombus wall deposits, a lack of venous valves and a characteristic shape of collateral vessels.

In all patients, serum creatinine was first documented within 24 h prior to MRI and was monitored once after MRI examination within 1 week after the application of the contrast medium.

The data were analyzed using descriptive statistical methods. For discrete and categorical data, the frequencies and relative frequencies were reported.

## 3. Results

**Figure 1:**
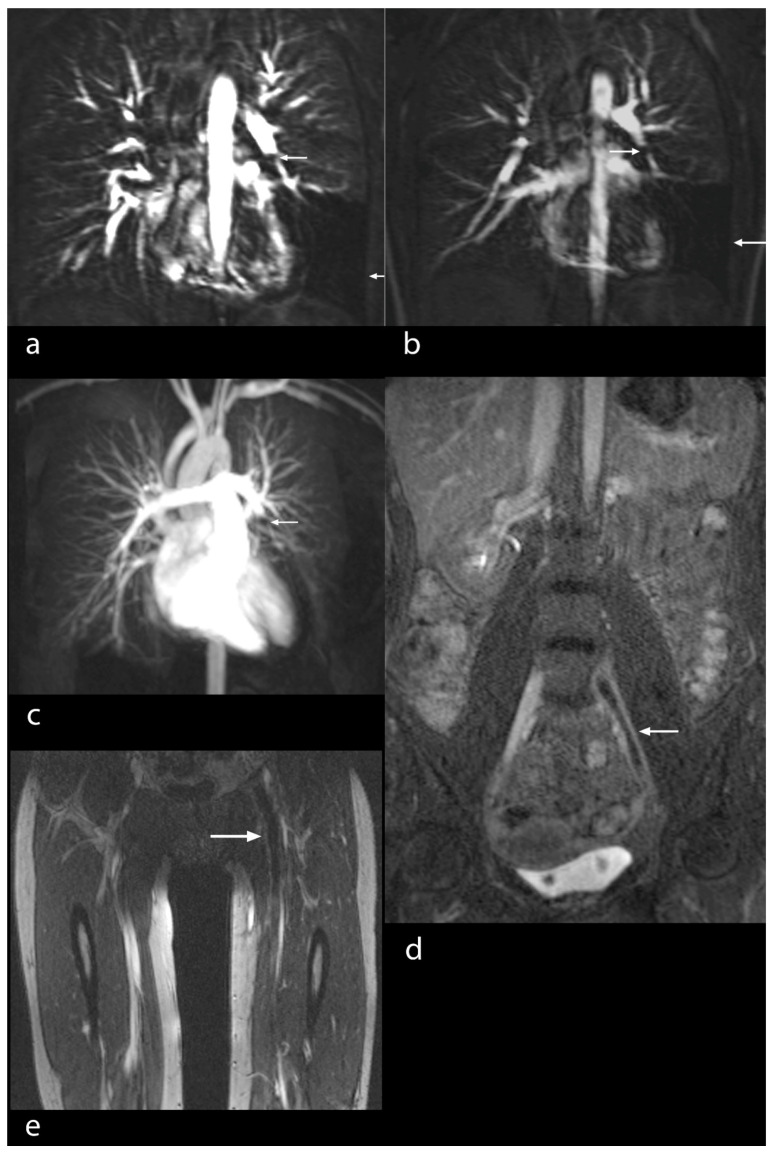
(**a**,**b**) Pulmonary MRA/MRV in a 20-year-old female patient under oral contraception and previously unrecognized factor V mutation. Thrombus in the left descending pulmonary artery (left arrow) with low enhancement in the capillary phase of the inferior pulmonary lobe (right arrow). Excellent examination quality; (**c**) 3D-MIP. Thrombus not visible. Total cut-off of the left pulmonary artery (see arrow). Perfusion deficit of the left inferior pulmonary lobe; (**d**,**e**) MRV, coronal. Thrombosis of the left iliac and the common femoral veins (see arrow). Good examination quality.

**Figure 2:**
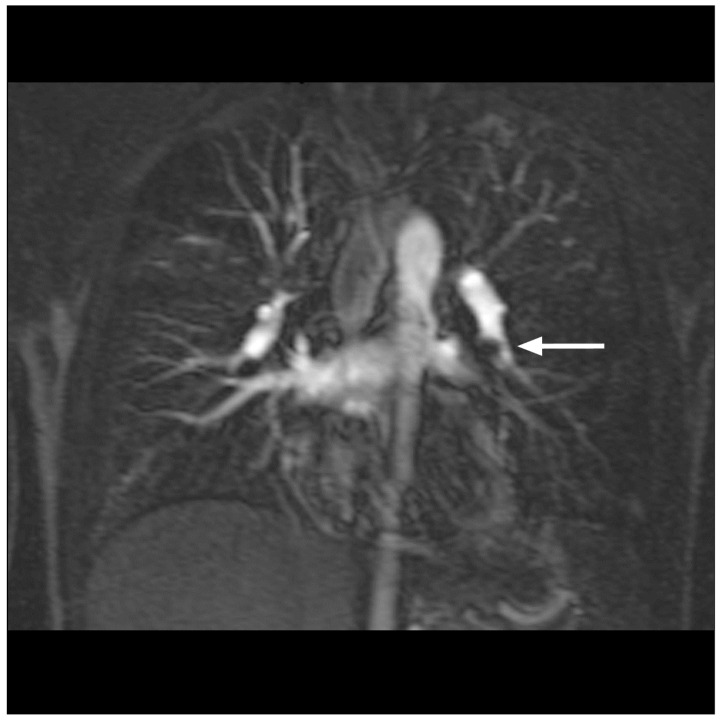
Pulmonary MRA in a 27-year-old male patient. Thrombus in the left pulmonary artery (see arrow) with hypoperfusion of the inferior pulmonary lobe and mild hypoperfusion in the right upper lobe. Good examination quality.

**Figure 3:**
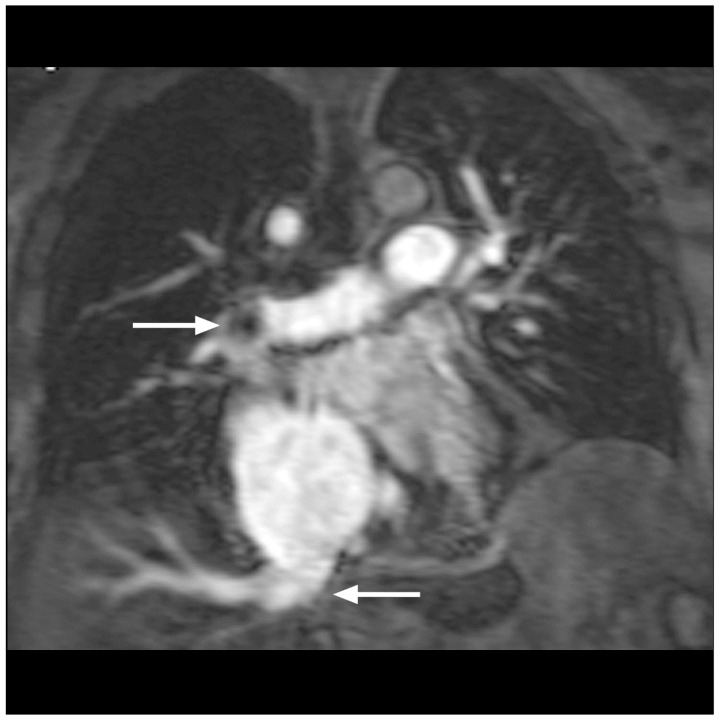
Pulmonary MRA in a 76-year-old male patient. After an initial CT examination and thrombolysis, the MRA control examination showed a residual central thrombus in the right pulmonary artery (upper arrow) with perfusion deficit. Right heart insufficiency with reflux of contrast medium in the inferior vena cava (lower arrow). Moderate examination quality.

**Figure 4:**
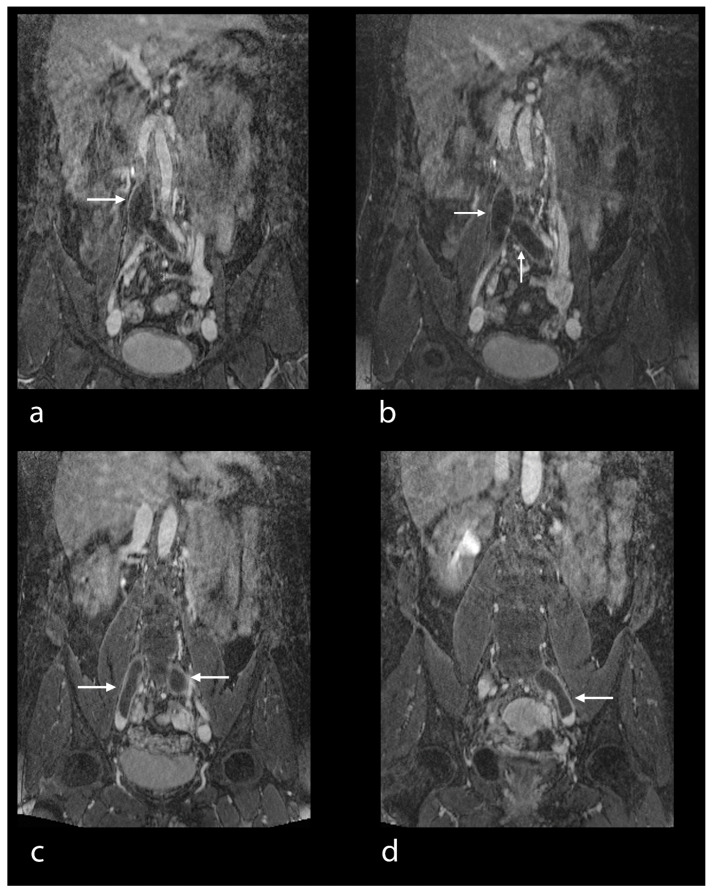
(**a**–**d**) MRV in a 47-year-old female patient. Thrombus in the inferior caval vein, the common iliac veins and the left external iliac vein (see arrows). Good examination quality.

Based on imaging quality, the definite diagnosis of PE was made in 88 patients by means of MRA/MRV. Four patients had two examinations, namely before and after filter implantation in the inferior caval vein. A total number of 92 studies were available for evaluation (100%). Image quality for the visualization of pulmonary arteries was excellent in 80 studies (87%) (Figure 1a–c), good in 6 studies (7%) (Figure 2), moderate in 5 studies (5%) (Figure 3) and poor in 1 study (1%). Image quality concerning peripheral veins was excellent in 83 examinations (90%) (Figure 4a–d), good in 8 examinations (9%) (Figure 1d,e) and poor in 1 examination (1%).

**Figure 5:**
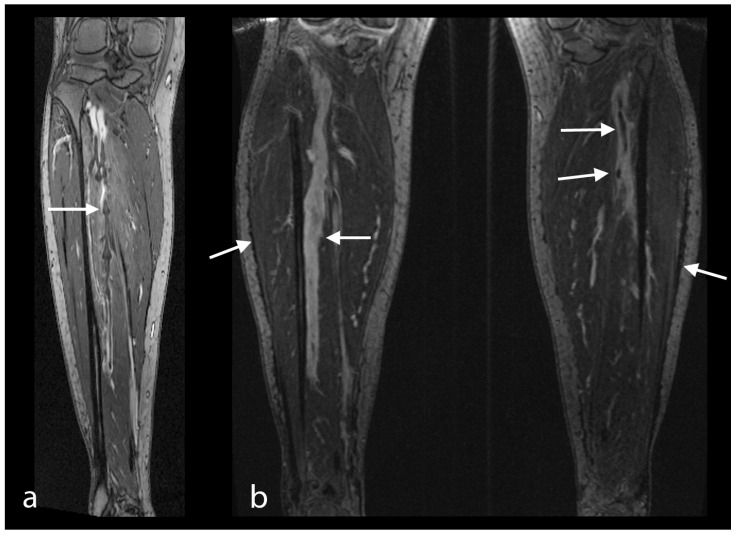
(**a**) MRV in a 26-year-old female patient. Recent thrombi in the right fibular veins, with partial obstruction in the distal segment of the popliteal vein. Excellent delineation of valves. Excellent examination quality; (**b**) MRV in a 44-year-old female patient. Minor PE after long distance flight. Left leg: filling defect representing acute thrombi in the distal popliteal and proximal fibular vein (see arrow). Right leg: venous wall irregularities, luminal inhomogeneities with destroyed venous valves, representing post-thrombotic changes (see upper arrows). Both legs: small subcutaneous fluid collections with global leg swelling (see arrows). Excellent examination quality.

The pulmonary arteries and their branches could be analyzed in detail based on good visualization due to the contrast agent (Figure 1a–c). The same was true for peripheral veins, including the inferior vena cava, the femoral veins and the deep veins of the calf in their entire length (Figure 1d,e, Figure 4a–d and Figure 5a,b).

The principal findings of MRI in PE were the direct visualization of intraluminal clotting as a filling defect and arterial occlusion with total cut-off of a vessel with the possible proximal enlargement of the vessel caliber (Figure 1a–c). However, for DVT, thrombi were visualized through a lack of intraluminal contrast filling (Figure 1, Figure 4 and Figure 5a,b). The following radiological features were considered characteristic for chronic thrombi: initial contrast enhancement of the thrombi, recanalization with absence of venous valves and wall deposit and filling defect and contour irregularity of the vessel wall as well as the shape of collaterals (Figure 5b). In 29 of the 92 MRI studies, no signs of PE and DVT were found (32% of the examinations). In 30 studies, PE was definitely diagnosed (33% of the examinations), in 42 studies, DVT was confirmed (46% of the examinations), while in 20 studies (22% of the examinations), both signs of PE and DVT were identified (Figure 1). For DVT, most thrombi were located in the common and superficial femoral, popliteal and fibular veins (Figure 5), but in 11 studies, thrombi were located in the inferior vena cava or in an iliac vein (12%) (Figure 1d,e and Figure 4). In 44 DVT studies, signs of acute as well as of chronic thrombi (48%) were found, whereas in 18 examinations, merely signs of acute thrombi (20%) were identified.

**Figure 6:**
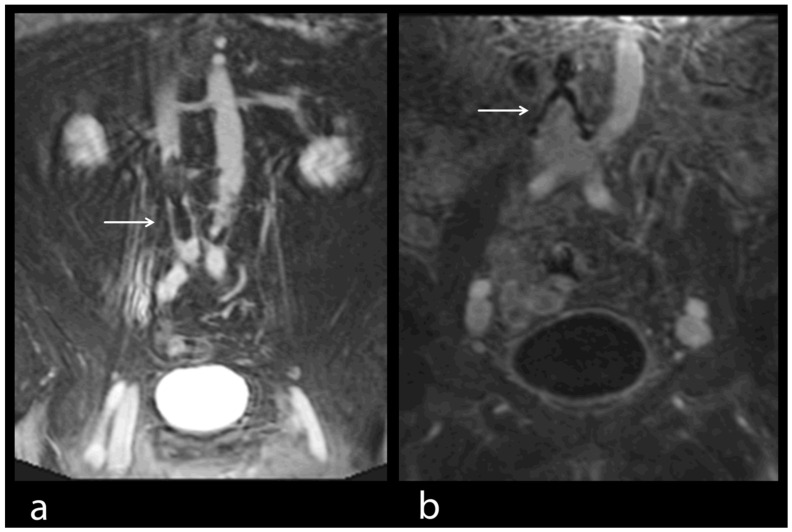
(**a**) MRV in a 32-year-old female patient. Optease^®^ filter (see arrow) with a captured thrombus at the follow-up examination. Good examination quality; (**b**) MRV in a 43-year-old male patient. Celect^®^ filter (see arrow) in the VCI to prevent further pulmonary embolism. Follow-up examination showed a thrombus-free filter, which could be removed successfully. Good examination quality.

Apart from the main diagnosis, further pathological findings, including pleural effusion, infarction pneumonia, pulmonary artery dilatation, dilatation of the right atrium and ventricle or regurgitation of the contrast media in the inferior vena cava (Figure 3), were seen and subsequently treated. In addition, a variety of important secondary diagnoses, specifically of mediastinal and abdominal lymphoma (three pat.), a bilioma (one pat.), a renal cell carcinoma (one pat.) and of a testicular tumor (one pat.), were made accidentally in the same examination without the extension of sequences. Thus, these patients not only were spared a second examination, but more importantly, immediate treatment was initiated. Furthermore, anatomic variants of vessels were detected, including renal vein anomalies (17 pat.), namely duplication, ring formation or retroaortic course, as well as the duplication of the inferior vena cava (1 pat.) and varicosity with insufficient perforating veins (23 pat.). We evaluated four patients for the implantation of an Optease^®^ (Cordis Corporation, Zug, Switzerland) (two pat.) and a Celect^®^ (Cook Medical Inc., Bloomington, IN, USA) (two pat.) vena cava filter (Figure 6a,b).

The Gd-containing contrast agent was well-tolerated by all patients. There were no moderate or severe adverse effects recognized. In particular, up to now, none of the patients has developed nephrogenic systemic fibrosis (NSF). The amount of Gdfos molecules regarding a single dose was smaller than in all established intravenous Gd-based MR contrast media. Compared to baseline values, the serum creatinine levels were not significantly elevated one week after MRA/MRV examination.

## 4. Discussion

Magnetic resonance angiography (MRA) has been evaluated for several years in suspected PE, but large-scale studies were published only recently [10,11]. Our current study, in addition, specifically considered the usefulness of filter implantation and explantation, and of stent planning. Although the feasibility of a combined protocol for the MRI diagnosis of DVT and PE has already been shown in a smaller number of patients, the current study was able to show the usefulness of this method for the planning of venous interventions, such as filter placement and retrieval, and for stent implantation.

In the most recently updated European Society of Cardiology (ESC) guidelines, the group concludes that this technique, although promising, is not yet ready for clinical practice due to its low sensitivity, high proportion of inconclusive MRA scans and low availability in most emergency settings [12]. We acknowledge that these techniques are best suited for high-volume sites that have a lot of experience running them, but that they may not be suitable for all sites. They can be used for an examination after treatment, and they are advantageous in young patients and in patients with reduced renal function [13]. Among our patients, 17% had reduced renal function with a creatinine level above 1.20 mg/dL and a glomerular filtration rate (eGFR) below 60 mL/min/1.73 m^2^ before MRA/MRV examination. None of these patients developed NSF. At present, the etiology of NSF has not been completely elucidated [13]. Gadofosveset trinatrium belongs to the group of linear and ionic agents which are considered to be less safe than the cyclic non-ionic agents now used in our institution. However, in our cohort, no renal function deterioration or other early or late side-effects were recognized. Specifically, the amount of gadolinium for one examination for a patient can be determined depending on the gadolinium-containing contrast agent used. Therefore, in order to achieve comparable image quality using Gadofosveset trinatrium, a 2- to 3-fold amount of gadolinium would have to be applied with the use of Gadovist^®^, e.g., due to rapid renal excretion of the non-blood pool agents.

We found only a minor proportion of inconclusive MRAs. Pulmonary MRA was also not recommended in the Prospective Investigation of Pulmonary Embolism Diagnosis III (PIOPED III) report due to technical difficulties at many sites resulting in non-diagnostic studies [14]. The results of this report raise the possibility that it might be used at sites with greater technical expertise [15]. Although it may be suggested that one should compare pulmonary MRA with the gold-standard CTA, we find it unethical to postpone treatment by performing a subsequent CTA study if definite conclusiveness can be reached with MRA at our institution. Vice versa, it would be unethical to perform MRA/MRV after CTA for scientific purposes. Therefore, we chose this study design. MRA images may still be limited in quality on the segmental level due to the reduced breath-holding capacity of certain patients, although the abdominal, pelvic and lower extremity venous images are of very good quality. However, infarction pneumonia and pleural effusion may also hint at the correct diagnosis of a recent PE. Furthermore, we know from previous studies that DVT and PE are very dynamic diseases. A late start of treatment bordering at 30 min. may result in an either slightly or an already dramatically increasing thrombus load in the peripheral venous system, in the VCI or in the pulmonary arteries. Additionally, the fibrinolytic activity of PE treatment is postponed. Performing a subsequent study in one patient would take at least 30 min. for transport and preparation only between the end of the CTA examination and the start of the following MRA exam. In the case of the use of regular contrast agent, such as Gadovist^®^ or Dotarem^®^ (unpublished data), the visual impression of a more distinct contrast enhancement of the vessel wall in fresh thrombosis is noted. Furthermore, an advantage of a blood pool agent is the use of a single dose, whereas, according to our experience, regular contrast agents are applied, depending on renal function, usually at least in double dose.

For the diagnosis of both PE as well as of DVT, contrast-enhanced MRA and MRV have proven to be superior methods to time-of-flight or phase-contrast MRI due to their sensitivity in the diagnosis of thromboembolic disease [16,17]. Our study demonstrates that a large number of vessels can be evaluated in a one-stop shop MRA/MRV which are otherwise difficult to examine, such as the pulmonary arteries, the inferior vena cava, the deep veins of the lower extremity or the deep pelvic veins, in the shortest amount of time with predominantly excellent image quality. Using ultrasound, particularly abdominal and pelvic vessels are difficult to examine due to the deep location of these vessels and due to artifacts, namely from intestinal gas or obesity [18]. All these drawbacks are not relevant to MRV [19,20]. Peripheral veins of the calf are not always depicted as a whole using other methods for examination, such as ultrasound or conventional phlebography, while MRV overcomes this limitation as well. Other conditions, including obesity, edema of extremities, wounds and pain, can additionally limit the evaluation using ultrasound and conventional phlebography. CTA is the standard method used to evaluate the pulmonary arteries, but radiation dose and to a lesser extent thyroid and renal function need to be considered [20]. The gold-standard methods result in major exposure (around 5–8 mSv for thorax, abdomen and pelvis, and further dependent on technique, equipment and patients’ size) to radiation and a high amount (80–120 mL) of nephrotoxic iodinated contrast media. Several studies report on the use of gadolinium MRA and phlebography for the diagnosis of PE. Different factors, including vascular opacification, motion artifacts, dosage and type of contrast agent, influence signal quality [21,22,23].

Gadofosveset enables data acquisition with high resolution in the first pass and a steady state. Furthermore, Gdfos can be used both dynamically and in the steady state. Both techniques are described in Table 1. In this preliminary study, both Gdfos-enhanced MRA and MRV demonstrated excellent imaging quality and can therefore be viewed as technically feasible, robust, effective methods and reliable diagnostic tools for VTE, including pulmonary embolism and deep venous thrombosis. The entire length and morphology of the vessel, irrespective of its location, anatomical variants, extent and aspect of the (acute or chronic) thrombus, as well as secondary pathologies, can be analyzed. Gdfos-enhanced MRA has already been shown to improve the imaging quality of the renal arteries compared to non-contrast MRA [24], due to intravascular enhancement while increasing spatial resolution, based on its reversible binding qualities to albumin. It also facilitates the examination of multiple vessels. It is well-tolerated by patients [24,25]. It has been applied in an expanding number of vascular regions and indications, including cerebral vessels, renal arteries, aortoiliac arteries, abdominal perforator vessels, lower extremity arteries, pedal arteries and in-stent alterations [26,27]. Gdfos-enhanced MRV is efficient in VTE [28].

The contrast-enhanced MRA of the pulmonary arteries is an alternative to CTA—as well as digital subtraction angiography—for the assessment of vascular morphology and function [15,19,29,30,31,32]. MRV can be used to accurately diagnose acute pelvic vein (including internal iliac vein and inferior vena cava) thrombosis better than CT and Doppler ultrasound [6,15,19,30,32]. While ultrasound may suffer from low resolution or because the patient is obese, CT suffers from needing radiation exposure. Furthermore, since hardware and software have improved, fewer limitations avert adequate examinations [12,33]. Non-enhanced MRA is a rather long protocol and produces many artefacts and is therefore considered an inadequate procedure. Gdfos-MRA has significantly improved sensitivity, specificity and accuracy compared to non-enhanced MRA or non-enhanced MRV [23]. The use of Gdfos could enhance the quality of the exam of the lower extremities and possibly the pulmonary angiogram.

Contrast-enhanced MRV helped evaluate the technical possibility and selection of the type of cava filter implantation (Figure 6a,b) together with finding the most appropriate route for filter implantation without further testing [12]. Furthermore, MRV facilitates the planning of stent implantation after pelvic vein thrombosis [17,34]. Prior to cava filter implantation, important questions concerning the optimal length and landing zone of the filter can be answered by CE-MRV. The number and the angle of inferior caval vein branches, as well as the length of the inferior caval vein distal of the branches, can be determined. Furthermore, an access site, free of significant thrombi, can be chosen. The diameter and circumference of the inferior caval vein can be exactly measured for the choice of the filter size.

As with any imaging modality, a series of different types of artifacts influence the interpretation and cause limitations of the method, such as motion and metal artefacts. Therefore, MIP and source images should be interpreted together [16,21]. The total examination time of the combined MR protocol was 10 min or less for each patient. Our mean door-to-door time was 15 min and depended mainly on the radiographer’s experience and the patients’ condition. The spatial resolution of MRA was increased by lowering the slice thickness and increasing the matrix size. Nevertheless, the breath hold time would then increase as well. Our experience in VTE in MRV with a double dose of 1 molar Gdfos contrast media led us to implement these parameters.

Possible limitations of this study are the preselection of the patients, because of the limited availability of examination time slots in MR. The accessibility of MRA/MRV for the diagnosis of VTE is still limited, and excellent interpretation skills are mandatory [35]. However, an increasing potential of MRA and MRV is recognized, especially in young patients, as well as in follow-up situations and for treatment planning to avoid radiation.

Our study merely focused on the early post-acute phase of PE/DVT and the applicability of MRA/MRV as a follow-up instrument. Many patients with primary pulmonary hypertension, termed genuine, may in fact be patients with chronic recurrent PE due to recurrent DVT. Furthermore, this modality was used in patients for decision making prior to cava filter implantation and retrieval (Figure 6a,b). We used a double dose of cyclic non-blood pool contrast agent to mimic Gdfos in order to achieve a similar vessel enhancement of all veins in the whole body. In comparison to former studies with the ultrasmall superparamagnetic iron oxide (USPIO) intravascular contrast agent NC100 150 Clariscan^®^ (Nycomed Imaging AS, Oslo, Norway), the depiction of thrombi, especially of the lower legs, seemed to be much better with Gdfos [33,36]. A possible reason may be the contrast enhancement of the inflamed venous vessel wall with Gdfos, because of better extravasal enhancement in comparison to Clariscan^®^. However, large thrombi, such as those that can be found in the external iliac veins, the common iliac veins and the inferior caval vein, seem to be equally well-identifiable with all contrast agents. Furthermore, we did not compare different contrast agents in this study to answer the question of which agent might be better to diagnose large thrombi. It seems that the visibility of large thrombi might be at least equal to Gdfos with the use of cyclic non-blood pool contrast agents at double dose. Although the management and treatment decisions [18] for an individual patient with VTE of various stages lie in the hands of both the clinician and the interventional radiologist, it is the responsibility of the radiologist to perform a precise diagnosis of the VTE stage and possible ensuing complications, since accurate diagnosis will have a definite impact on the prognosis and the patient’s quality of life in the future. For example, the accurate diagnosis of large thrombi (of up to 8 mm in diameter in fibular veins and internal iliac veins) has an impact on the risk of further life-threatening PE, especially in patients with chronic pulmonary artery hypertension.

## 5. Conclusions

The one-stop shop, blood pool, contrast-enhanced MRA/MRV proved to be a robust, effective method and a rapid tool for the diagnosis of venous thromboembolic disease. Both PE and DVT were visible with predominantly excellent qualities with important implications for the treatment planning of the patient. Furthermore, the accurate planning of venous invasive procedures, such as venous catheter interventions, specifically thrombus retrieval and stent implantation as well as MRV-guided cava filter implantation and retrieval, were facilitated. These data contribute to highlighting the potential role that this radiation free technique could have in this clinical context, particularly in young patients or patients with impaired renal function. However, further data coming from large-scale studies are still needed to reinforce the use of this modality in daily clinical practice for the diagnosis of VTE.

## Figures and Tables

**Table 1:** Parameters used for pulmonary MR angiography and MR venography.

	TR (ms)	TE (ms)	Flip Angle (Degree)	THK (mm)	FOV	Matrix	Duration (s)
PMRA	2.4	1.04	20	3	400–500	320 × 240	20 (4 dynamic sequences)
MRV * (3FOV)	4.3	1.34	34	1.5	450–500	320 × 256	21,20,24
HR-MRV (3FOV)	6.21	2.08	24	1.3–1.1	500	384 × 384–512 × 512	123,225,286

Legend: HR = high resolution; MRV = MR venography; PMRA = pulmonary MR angiography. * Only in non-compliant patients.

## Data Availability

The data presented in this study are available on request from the corresponding author.

## References

[1] Andia M.E., Saha P., Jenkins J., Modarai B., Wiethoff A.J., Phinikaridou A., Grover S.P., Patel A.S., Schaeffter T., Smith A. (2014). Fibrin-targeted magnetic resonance imaging allows in vivo quantification of thrombus fibrin content and identifies thrombi amenable for thrombolysis. Arter. Thromb. Vasc. Biol..

[2] Jaff M.R., McMurtry M.S., Archer S.L., Cushman M., Goldenberg N., Goldhaber S.Z., Jenkins J.S., Kline J.A., Michaels A.D., Thistlethwaite P. (2011). Management of massive and submassive pulmonary embolism, iliofemoral deep vein thrombosis, and chronic thromboembolic pulmonary hypertension: A scientific statement from the American Heart Association. Circulation.

[3] Hochegger B., Ley-Zaporozhan J., Marchiori E., Irion K., Soares Souza A., Moreira J., Kauczor H.U., Ley S. (2011). Magnetic resonance imaging findings in acute pulmonary embolism. Br. J. Radiol..

[4] Obernosterer A., Aschauer M., Portugaller H., Köppel H., Lipp R.W. (2005). Three-dimensional gadolinium-enhanced magnetic resonance angiography used as a “one-stop shop” imaging procedure for venous thromboembolism: A pilot study. Angiology.

[5] Jiménez D., Rodríguez C., León F., Jara–Palomares L., López-Reyes R., Ruiz-Artacho P., Elías T., Otero R., García-Ortega A., Rivas-Guerrero A. (2021). Randomised controlled trial of a prognostic assessment and management pathway to reduce the length of hospital stay in normotensive patients with acute pulmonary embolism. Eur. Respir. J..

[6] Winer-Muram H.T., Gurney J.W. (2006). Pulmonary Emboli. Diagnostic Imaging Chest, Part II, Section 4: Pulmonary Vasculature.

[7] Liberman A.L., Daruwalla V.J., Collins J.D., Maas M.B., Botelho M.P., Ayache J.B., Carr J., Ruff I., Bernstein R.A., Alberts M.J. (2014). Diagnostic yield of pelvic magnetic resonance venography in patients with cryptogenic stroke and patent foramen ovale. Stroke.

[8] Blättler W., Gerlach H., Hach-Wunderle V., Konstantinides S., Noppeney T., Pillny M., Riess H., Schellong S., Stiegler H., Wildberger J.E. (2010). Bein- und Beckenvenenthrombose (TVT). Vasa Suppl..

[9] Meany J.F.M., Ridgway J.P., Chakraverty S., Robertson I., Kessel D., Radjenovic A., Kouwenhoven M., Kassner A., Smith M.A. (1992). Stepping-table gadolinium-enhanced digital subtraction MR angiography of the aorta and lower extremity arteries: Preliminary experience. Radiology.

[10] Hansch A., Betge S., Poehlmann G., Neumann S., Baltzer P., Pfeil A., Waginger M., Boettcher J., Kaiser W.A., Wolf G. (2011). Combined magnetic resonance imaging of deep venous thrombosis and pulmonary arteries after a single injection of a blood pool contrast agent. Eur. Radiol..

[11] Tsuchiya N., van Beek E.J.R., Ohno Y., Hatabu H., Kanczor H.U., Swift A., Vogel-Claussen J., Biederer J., Wild J., Wielpütz M.O. (2018). Magnetic resonance angiograpy for the primary diagnosis of pulmonary embolism: A review from the international workshop for pulmonary functional imaging. World J. Radiol..

[12] Konstantinides S.V., Meyer G., Becattini C., Bueno H., Geersing G.-J., Harjola V.-P., Huisman M.V., Humbert M., Jennings C.S., Jiménez D. (2020). 2019 ESC Guidelines for the diagnosis and management of acute pulmonary embolism developed in collaboration with the European Respiratory Society (ERS). Eur. Heart J..

[13] Michaely H.J., Aschauer M.A., Deutschmann H., Bongartz G., Gutberlet M., Woitek R., Ertl-Wagner B., Kucharczyk W., Hammerstingl R., De Cobelli F. (2017). Gadobutrol in renally impaired patients—Results of the GRIP study. Investig. Radiol..

[14] Stein P.D., Chenevert T.L., Fowler S.E., Goodman L.R., Gottschalk A., Hales C.A., Hull R.D., Jablonski K.A., Leeper KVJr Naidich D.P., Sak D.J. (2010). Gadolinium-enhanced magnetic resonance angiography for pulmonary embolism: A multicenter prospective study (PIOPED III). Ann. Intern. Med..

[15] Fu Q., Liu D., Kong X., Lei Z. (2020). Combined MR imaging for pulmonary embolism and deep venous thrombosis by contrast-enhanced MR volume interpolated body examination. Curr. Med. Sci..

[16] Ruehm S.G., Schneider G., Prince M.R., Meaney J.F.M., Ho V.B. (2005). MR Venography. Magnetic Resonance Angiography.

[17] Zollikofer C.L., Antonucci F., Stuckmann G., Mattias P., Brühlmann W.F., Salomonowitz E.K. (1992). Use of Wallstent in the venous system including hemodialysis-related stenoses. Cardiovasc. Interv. Radiol..

[18] Leiner T., Kessels A.G., Nelemans P.J., Vasbinder G.B., de Haan M.W., Kitslaar P.E., Ho K.Y., Tordoir J.H., van Engelshoven J.M. (2005). Peripheral arterial disease: Comparison of color duplex US and contrast-enhanced MR angiography for diagnosis. Radiology.

[19] Torbicki A., Perrier A., Konstantinides S., Agnelli G., Galiè N., Pruszczyk P., Bengel F., Brady A.J., Ferreira D., Janssens U. (2008). Guidelines on the diagnosis and management of acute pulmonary embolism. The task force for diagnosis and management of acute pulmonary embolism of the European Society of Cardiology (ESC). Eur. Heart J..

[20] Sostman D.H., Jablonski K.A., Wooddard P.K., Stein P.D., Naidich D.P., Chenevert T.L., Weg J.G., Hales C.A., Hull R.D., Goodman L.R. (2012). Factors in the technical quality of gadolinium enhanced magnetic resonance angiography for pulmonary embolism in PIOPED III. Int. J. Cardiovasc. Imag..

[21] Woodard P.K., Chenevert T.L., Dirk Sostman H., Jablonski K.A., Stein P.D., Goodman L.R., Londy F.J., Narra V., Hales C.A., Hull R.D. (2012). Signal quality of single dose gadobenate dimeglumine pulmonary MRA examinations exceeds quality of MRA performed with double dose gadopentetate dimeglumine. Int. J. Cardiovasc. Imag..

[22] Wagner M., Rief M., Asbach P., Vogtmann T., Huppertz A., Beling M., Butler C., Laule M., Warmuth C., Taupitz M. (2010). Gadofosveset trisodium-enhanced magnetic resonance angiography of the left atrium—A feasibility study. Eur. J. Radiol..

[23] Goyen M. (2008). Gadofosveset-enhanced magnetic resonance angiography. Vasc. Health Risk Manag..

[24] McGregor R., Vymazal J., Martinez-Lopez M., Neuwirth J., Salgado P., Beregi J.P., Peduto A., de la Pena-Almaguer E., Slater G.J., Shamsi K. (2008). A multi-center, comparative, phase 3 study to determine the efficacy of gadofosveset-enhanced magnetic resonance angiography for evaluation of renal artery disease. Eur. J. Radiol..

[25] Schneider G., Pasowicz M., Vymazal J., Seidl Z., Aschauer M., Konopka M., Bilecen D., Iezzi R., Ballarati C. (2010). Gadobenate Dimenglumine and Gadofosveset Trisodium for MR Angiography of the renal arteries: Multicenter intraindividual crossover comparison. AJR Am. J. Roentgenol..

[26] Vogt F.M., Herborn C.U., Parsons E.C., Barkhausen J., Kröger K., Goyen M. (2007). Diagnostic performance of contrast-enhanced angiography of the aortoiliac arteries with the blood pool agent Vasovist: Initial results in comparison to arterial DAS. Rofo.

[27] Hecht E.M., Rosenkrantz A. (2009). Pulmonary MR angiography techniques and applications. Magn. Reson. Imaging Clin. N. Am..

[28] Ley S., Ley-Zaporozhan J., Pitton M.B., Schneider J., Wirth G.M., Mayer E., Düber C., Kreitner K.F. (2011). Diagnostic performance of state-of-the-art imaging techniques for morphology assessment of vascular abnormalities in patients with chronic thromboembolic pulmonary hypertension (CTEPH). Eur. Radiol..

[29] Haage P., Krimgs T., Schmitz-Rode T. (2002). Nontraumatic vascular emergencies: Imaging and intervention in acute venous occlusion. Eur. Radiol..

[30] Enden T., Storås T.H., Negård A., Haig Y., Sandvik L., Gjesdal K.I., Sandset P.M., Kløw N.E. (2010). Visualization of deep veins and detection of deep vein thrombosis (DVT) with balancec turbo field echo (b-TFE) and contrast-enhanced T1 fast field echo (CE-FFE) using a blood pool agent (BPA). J. Magn. Reson. Imaging.

[31] Schaefer-Prokop C., Prokop M. (2005). MDCT for the diagnosis of acute pulmonary embolism. Eur. Radiol..

[32] Fink C., Kauczor H.U. (2009). MR Angiography of the Pulmonary Vasculature. MRI of the Lung.

[33] Aschauer M., Deutschmann H.A., Stollberger R., Hausegger K.A., Obernosterer A., Schöllnast H., Ebner F. (2003). Value of a blood pool contrast agent in MR venography of the lower extremities and pelvis: Preliminary results in 12 patients. Magn. Reson. Med..

[34] Kucher N., Stuck A.K. (2015). CardioPulse: Interventional treatment of venous thromboembolism: A review and update of treatments in 2014. Eur. Heart J..

[35] Prince M.R., Sostman H.D. (2003). MR Venography: Unsung and Underutilized. Radiology.

[36] Mathevosian S., Takegawa Y., Hassani C., Jalili M.H., Finn J.P., Yuan C., Li D. (2022). Evaluation of aortic stent endoleaks using ferumoxytol enhanced MR angiography. Proceedings of the 34th Annual International Conference of SMRA.

